# Loop-mediated isothermal amplification-lateral-flow dipstick (LAMP-LFD) to detect *Mycoplasma ovipneumoniae*

**DOI:** 10.1007/s11274-019-2601-5

**Published:** 2019-01-30

**Authors:** Jie Zhang, Junjun Cao, Mingsong Zhu, Mingguo Xu, Feng Shi

**Affiliations:** 1 College of Life Sciences, Shihezi University, Xin Jiang, Shihezi, 832000 China; 2 College of Animal Science and Technology, Shihezi University, Xin Jiang, Shihezi, 832000 China

**Keywords:** *Mycoplasma ovipneumoniae*, Elongation factor Tu gene (*EF-TU*), Loop-mediated isothermal amplification, Lateral flow dipstick, Visual detection

## Abstract

In order to establish a rapid detection method for *Mycoplasma ovipneumoniae*, this study used the loop-mediated isothermal amplification (LAMP) technique to carry out nucleic acid amplification and chromatographic visualization via a lateral flow dipstick (LFD) assay. The *M. ovipneumoniae* elongation factor *TU* gene (*EF-TU*) was detected using a set of specific primers designed for the *EF-TU* gene, and the *EF-TU* FIP was detected by biotin labeling, which was used in the LAMP amplification reaction. The digoxin-labeled probe specifically hybridized with LAMP products, which were visually detected by LFD. Here, we established the *M. ovipneumoniae* LAMP-LFD rapid detection method and tested the specificity, sensitivity, and clinical application of this method. Results showed that the optimized LAMP performed at 60 °C for 60 min, and LFD can specifically and visually detect *M. ovipneumoniae* with a minimum detectable concentration at 1.0 × 10^2^ CFU/mL. The sensitivity of LAMP-LFD was 1000 times that of the conventional PCR detection methods, and the clinical lung tissue detection rate was 86% of 50 suspected sheep infected with *M. ovipneumoniae*. In conclusion, LAMP-LFD was established in this study to detect *M. ovipneumoniae*, a method that was highly specific, sensitive, and easy to operate, and provides a new method for the prevention and diagnosis of *M. ovipneumoniae* infection.

**Electronic supplementary material:**

The online version of this article (10.1007/s11274-019-2601-5) contains supplementary material, which is available to authorized users.

## Introduction

*Mycoplasma ovipneumoniae* (*M. ovipneumoniae*) is an important pathogen that causes atypical pneumonia in goats and sheep (Nicholas et al. 2002; Parham et al. 2006; Xin et al. 2012; Besser et al. 2013). In recent years, *M. ovipneumoniae* infection has been reported in many regions of China, with gradually increasing mortality rates (Handeland et al. 2014; Besser et al. 2014). Sheep infected with *M. ovipneumoniae* show respiratory disorders, runny noses, weight loss, growth retardation, and primary infection within 1–3 months, depending on the age of the sheep (Besser et al. 2017). The control and prevention of *M. ovipneumoniae* is difficult and has caused great economic losses to the sheep industry in many countries (Giangaspero et al. 2012; Xue et al. 2017). In addition, upon *M. ovipneumoniae* infection, sheep become susceptible to other diseases. Therefore, there is an urgent need to develop a rapid and accurate method to detect *M. ovipneumoniae*. Such a method not only provides a reference for early prevention, diagnosis, and epidemiological investigation but also has a certain core value for farmers.

At present, the main methods for detecting *M. ovipneumoniae* are pathogen diagnosis, enzyme linked immunosorbent assay (ELISA), and PCR (Jiang et al. 2016) (Jiang et al. 2016; Ziegler et al. 2014; Li et al. 2016; Yang et al. 2014; Kılıc et al. 2013). Furthermore, the research of *M. ovipneumoniae* has mainly concentrated on the isolation, identification, and detection technology of pathogenic bacteria (Butler et al. 2017). Although pathogen separation is the “gold standard” for *M. ovipneumoniae* testing, these steps are relatively tedious and time-consuming and can no longer meet the requirements of animal husbandry. Immunological methods mainly rely on the specific reaction between antigen and antibody, and while the sensitivity and specificity of these methods are high, these methods are relatively complex and rely on assorted experimental conditions and other factors; thus, their clinical application is limited. Furthermore, molecular detection methods require expensive laboratory instruments and manipulation, which may not be readily available in remote areas. In 2000, Notomi et al. developed a loop-mediated isothermal amplification (LAMP), which has the advantages of high sensitivity, good specificity, and simple operation (Domesle et al. 2017; Chen et al. 2017). The LAMP amplification principle is based on the use of 4 primers that are designed for 6 specific regions of the target gene using the strand displacement characteristics of *Bst* DNA polymerase (Velders et al. 2018; Feng et al. 2017; Zheney et al. 2018; Sheu et al. 2018). At certain temperatures, the 4 primers identify 6 specific regions of the target gene and continuously the process of extension replacement. Therefore, use of the appropriate primer is very important for complete LAMP amplification. In addition to focusing on the highly specific identification of target genes at 6 regions, the annealing temperatures of the inner primers F2 (B2) and F1c (B1c) should be higher than those of the outer primers to ensure that the inner primers appropriately bind the target gene pairs. In order to improve the specificity and sensitivity of the LAMP reaction, 1 pair of ring primers can be designed between the primers and the internal and external primers. LAMP can achieve rapid amplification of nucleic acids in a simple constant-temperature device, and the amplification products can be detected using a turbidimeter, electrophoresis, and by adding fluorescent dye into the tube reaction (Park et al. 2017). LAMP detection time is about 60 min, compared with the conventional PCR method; thus it not only reduces reaction time but also increases sensitivity and specificity, with good prospects and development potential. However, LAMP amplification products need special gel-imaging equipment and other instruments through electrophoresis analysis. During the experiment, it is necessary to contact carcinogen EB, and the fluorescent reagent used in fluorescence detection is expensive, which limits the application and popularization of the technology in grass-roots units. The LAMP-LFD method uses a biotin LAMP product hybridized with a digoxin-labeled DNA probe that is complexed with a gold-labeled anti-digoxin antibody. This hybridization product is trapped by a biotin ligand and bound to a lateral flow test strip, forming an immune complex. Non-hybridized digoxin-labeled probes pass through the test line (T) and bind to the sheep anti-mouse immunoglobulin G (IgG) antibody control line (C). LAMP-LFD is a LAMP amplification detection technology, wherein the product of LAMP is detected on a lateral flow test strip by color to determine the experimental results. This method removes dependence on equipment and avoids contact with ethidium bromide. The whole reaction is based on LAMP detection, which can be completed within 5–10 min; therefore, it has good prospects for application.

So far, many researchers have reported the use of LAMP-LFD technology to detect pathogenic bacteria (Lalle et al. 2018; Wachiralurpan et al. 2017; Kongkasuriyachai et al. 2017; Huang et al. 2017), but the use of LAMP to detect *M. ovipneumoniae* is still lacking. Based on the *M. ovipneumoniae EF-TU* gene, we designed 6 LAMP primers and 1 digoxin-labeled probe to optimize LAMP detection conditions. Then LFD technology was added to establish an accurate and efficient *M. ovipneumoniae* LAMP-LFD assay. For some small- and medium-sized farms and scattered farmers, especially in remote areas, a fast, practical, sensitive and accurate way to diagnose pneumonia caused by *M. ovipneumoniae* is established in this study, which may be useful for improving economic conditions.

## Materials and methods

### Strains and samples

*Escherichia coli, Staphylococcus aureus, Salmonella pullorum, Mycoplasma bovis* (*M. bovis*), *Mycoplasma hyopneuminiae* (*M. hyopneuminiae*) and *Mycoplasma mycoides* subsp. *Capri* (*M. mycoides* subsp. *Capri*) were preserved by the laboratory of Microbiology Teaching and Research at Shihezi University, and *M. ovipneumoniae* strain Y98 was purchased from the China Institute of Veterinary Drugs Control. Twenty *M. ovipneumoniae-*infected sheep lung tissue were collected from the sheep farms in the surrounding areas of Manasi and Shihezi, and were stored at − 80 °C.

### DNA extraction

DNA was extracted from strains and sheep lung tissue according to the instruction of Genomic DNA Extraction Kit (Sangon Biotech Co., Ltd, Shanghai, China). The extracted genomic DNA was evaluated on a Nanodrop and its concentration was calculated, we used ten-fold serial dilutions with sterile water to dilute all Genomic DNA to approximately 10^1^–10^7^ CFU/mL, and stored at − 20 °C until use.

### Design of primers and probes

**Fig. 1:**
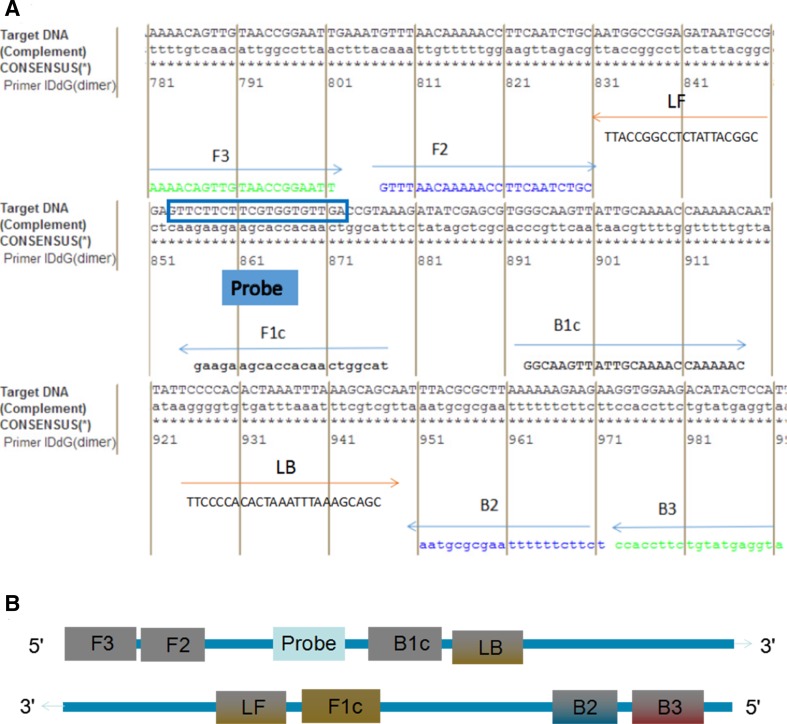
LAMP primer design for *M. ovipneumoniae EF-TU* gene. **A** The location of *M. ovipneumoniae EF-TU* primers and probes in the sequence. **B** The location of *M. ovipneumoniae EF-TU* primers and probes

Primers were designed according to the *M. ovipneumoniae EF-TU* gene published in GenBank in NCBI (NO: JQ990999) combined with the LAMP primer design principle. The Primer Explorer V3 (http://primerexplorer.Jp; Eiken Chemical Co., Ltd., Tokyo, Japan) online Primer design software was used to screen out a set of specific good primers and probes. In addition, Primer 5.0 software was used to design the Primer *EF-TU*F and *EF-TU*R (Table 1; Fig. 1), and the expected amplification fragment size was 1209 bp.

**Table 1:** The specific primers and probes designed for *M. ovipneumoniae* detection

Name of primers	Type	Sequences (5′→3′)	Sizes of amplicons (bp)
PCR			
*EF-TU*F	Forward prime	ATGGCAGTTGTTAAAACTGGTG	1209
*EF-TU*R	Backward primer	TTATTTAATAATTTCAGTTACTGTTCC	
LAMP			
*EF-TU*F3	Forward-outer prime	AAAACAGTTGTAACCGGAATT	211
EF-TUB3	Backward-outer primer	ATGGAGTATGTCTTCCACC	
*EF-TU*FIP^a^ (F1c + TTTT + F2)	Forward-inner primer	Biotin -TACGGTCAACACCACGAAGAAGTTTTGTTTAACAAAAACCTTCAATCTGC	165
*EF-TU*BIP (B1c + TTTT + B2)	Backward-inner primer	GGCAAGTTATTGCAAAACCAAAAACTTTTTCTTCTTTTTTAAGCGCGTAA	
LB	Loop-backward primer	TTCCCCACACTAAATTTAAAGCAGC	117
LF	Loop-forward primer	CGGCATTATCTCCGGCCATT	
Probe for LAMP-LFD *EF-TU*-HP^b^	Hybridization probe	Digoxin -GTTCTTCTTCGTGGTGTTGA	/

^a^5′-Labeled with biotin when used in the LAMP-LFD assay

^b^5′-Labeled with digoxin when used in the LAMP-LFD assay

### LAMP assay

*Mycoplasma ovipneumoniae* genomic DNA was extracted as a positive template, with 25 µL for the total reaction system comprising of the following: 10× ThermoPol Buffer 2.5 µL, MgSO_4_ (25 mmol/L) (Sigma, MO, USA) 1.5 µL, dNTPs (10 mmol/L) (Sangon Biotech Co., Ltd, Shanghai, China) 3.5 µL, Bst 2.0 DNA polymerase (8 U/L) (New England Biolabs, Ltd, Beijing, China) 1 µL, 3 µL primers (including inner primers (20 mol/L), outer primers (10 mol/L) and loop primers (15 mol/L) 1 µL), 2.5 µL betaine (10 mol/L) (Sigma, MO, USA), 2 µL positive template, calcein (Sigma, MO, USA) 1 µL, ddH_2_O. Sterile water was used as the template in the negative control sample.

### Optimization of reaction temperature and time of the LAMP assay

The PCR tube into the constant temperature water bath was set to 58 °C–62 °C (increasing to 1 °C interval) reaction for 60 min. The temperature was increased to 80 °C for 5 min to inactivate the enzyme, and amplification products (7 µL) were run on 3% agarose gel electrophoresis to determine the optimal temperature. Meanwhile, the reaction time was optimized and set for 30–80 min (increasing in 10 min interval) at constant temperature water bath. After that, the amplified products (7 µL) were analyzed on 3% agarose gel electrophoresis, and the best reaction time was determined.

### Optimization of the internal and external primer concentration ratio of the LAMP assay

According to the best reaction temperature and time, the ratio of internal and external primers was set up according to 25 µL system: 1:1, 2:1, 3:1 and 4:1, respectively, and LAMP was amplified. The amplified products of 7 µL were determined by 3% agarose gel electrophoresis, and the best ratio of internal and external primers was determined.

### Optimal reaction system for the LAMP assay

The 25 µL system was set up, respectively by optimum reaction temperature, time, and the ratio of inner and outer primers, optimization of Mg^2+^ volume (1.0 µL, 1.5 µL, 2.0 µL, 2.5 µL) and dNTPs volume (1.0 µL, 1.5 µL, 2.0 µL, 2.5 µL), and 7 µL amplification products were run on 3% agarose gel electrophoresis to determine the best reaction system.

### LAMP-LFD assay

In the process of hybridization, LAMP amplification products of biotin labeling, and *EF-TU*-HP-specific hybridization formed biotin and digoxin probe amplification products. The amplification products were added to the sample pad of the LFD strip and migrated by capillary action, where the biotin LAMP product hybridized with a digoxin-labeled DNA probe and complexed with a gold-labeled anti-digoxin antibody. This hybridization product was trapped by a biotin ligand and bound to a lateral flow test strip, forming an immune complex bound to the test line (T). Non-hybridized digoxin-labeled probes passed through the test line (T) and bound to the sheep anti-mouse IgG antibody control line (C) (Extended Data Fig. S1). The experimental procedure and determination method were as follows: 5 µL LAMP amplification product was added to 80 µL buffer, the mixture was added to the sample mat on the test paper strip, and the result was read after 5–10 min. The lateral flow dipstick (LFD) was provided by the Nucleic Acids Labeling and Detecting Lab, Key Laboratory of Ministry of Education with Provincial Co-construction of Local and Ethnic High Incidence in Xinjiang (Shihezi, Xinjiang, China).

### The specificity and repeatability of LAMP-LFD

According on the above LAMP reaction conditions and system optimization, DNA was extracted from *M. ovipneumoniae, Escherichia coli, Staphylococcus aureus, Salmonella pullorum, M. Bovis, M. hyopneuminiae, M. mycoides subsp. Capri* and amplified. The product was evaluated with 3% agarose gel electrophoresis and LFD assay was performed. The reaction was repeated at least 3 times for LAMP-LFD verification.

### Sensitivity of LAMP-LFD

Sensitivity of LAMP-LFD was investigated with different concentrations of *M. ovipneumoniae* genomic DNA as template and sterile water as negative control, LAMP amplification was carried out using the reaction system and reaction conditions of the above optimization. Meanwhile, the PCR reaction was conducted with *EF-TU*F and *EF-TU*F as the upstream and downstream primers, and the two methods were compared. The PCR reaction system was 25 µL, comprising of 2xES Taq MasterMix 12.5 µL, upstream and downstream primer *EF-TU*F and *EF-TU*F, respectively 0.4 µL, positive template 2 µL, and ddH_2_O. The PCR reaction procedure was 95 °C for 5 min; 30 cycles of 94 °C for 30 s, 55 °C for 30 s, and 72 °C for 80 s and a final extension of 72 °C for 10 min. The amplification products were tested by electrophoresis and LFD test strips respectively, and the detection sensitivity was the lowest detection concentration of the system.

### Clinical application and detection of *M. ovipneumoniae*

#### Pathogen separation method detection (gold standard)

Take 0.5 g of lung tissue that suspected *M. ovipneumoniae*-infected sheep lesions, grind it into minced under sterile conditions, and inoculate the grinded tissue fluid 1:10 in Mo liquid medium and add appropriate amount of ampicillin at 37 °C. Cultured at 5 °C and 5% CO_2_ for 4–5 days. Passaged when the medium became orange or yellow. Passaging bacteria were inoculated on solid medium after 3 passages, cultured for 7–8 days, and observed papillary colonies under an inverted microscope, and statistical results. If there is a papillary colony, a single colony is picked and inoculated in a liquid culture medium. After the color of the culture medium turns yellow, PCR is performed using Mo-specific primers, and the results are statistically analyzed.

In order to test the feasibility of the established LAMP-LFD assay, we established a reference standard that combined the results of the pathogen separation method and PCR were used to detect the lung tissues of 50 suspected *M. ovipneumoniae*-infected positive sheep, and the three assays were compared.

## Results

### Optimization of reaction temperature and time of LAMP assay

As detailed in the Methods section, separate experiments were carried out to determine the ideal temperature and reaction time for the LAMP assay. LAMP reaction had the brightest and showed clear trapezoid strip at 60 °C and 60 min (Extended Data Fig. S2A, B). This indicated that LAMP method detection of *M. ovipneumoniae* only required 60 min, which was faster than PCR detection.

### Optimization of the internal and external primer concentration ratio for the LAMP assay

As outlined in the Methods section, the best ratio of internal and external primers (1:1, 2:1, 3:1, and 4:1) was determined once the optimal temperature and reaction time were determined. As shown in Extended Data Fig. S3, the 3:1 ratio of internal and external primers had the best amplification product.

### Optimal reaction system of LAMP assay

Upon optimizing the reaction temperature, time, and the ratio of inner and outer primers, we next optimized the MgSO_4_ volume (1.0 µL, 1.5 µL, 2.0 µL, 2.5 µL) and dNTPs volume (1.0 µL, 1.5 µL, 2.0 µL, 2.5 µL), and 7 µL amplification products were run on 3% agarose gel electrophoresis to determine the best system. The results showed that 2 µL MgSO_4_ is (Extended Data Fig. 4A) and 2.5 µL dNTPs (Extended Data Fig. 4B) resulted in the highest LAMP amplification.

Reaction parameters were also optimized by single factor optimization experiment to determine the optimal reaction system of 25 µL as follows: 2.5 µL 10× ThermoPol buffer, 2.0 µL MgSO_4_, 3 µL, 2 µL primers (including internal and external 3:1 in primers and loop primer for 1 µL), 2.5 µL dNTPs, 2.5 µL betaine (10 mol/L), 1 µL Bst 2.0 DNA polymerase, 2 µL positive template, 1 µL calcein, and ddH_2_O. The PCR tube was placed in a constant temperature water bath at 60 °C for 60 min, 80 °C 5 min, and the enzyme was inactivated.

### The establishment of the *M. ovipneumoniae* LAMP-LFD assay

**Fig. 2:**
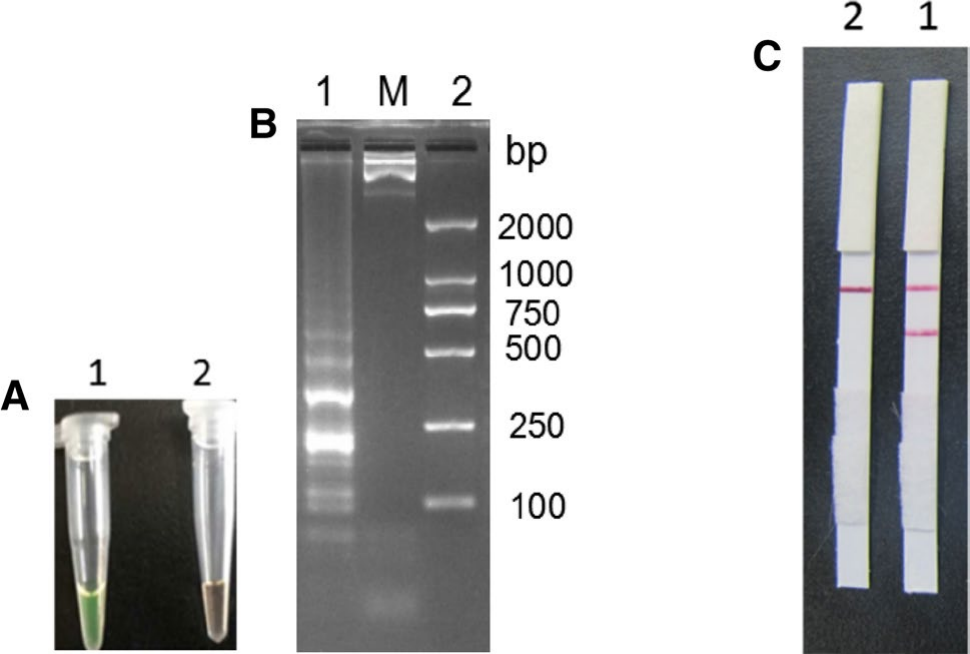
The establishment of *M. ovipneumoniae* LAMP-LFD assay. M: DNA Marker; 1: LAMP products; 2: Negative control. **A** calcein-visual LAMP amplification product. **B** Electrophoretic analysis of LAMP amplification products. **C** LAMP-LFD detection result

*Mycoplasma ovipneumoniae* genomic DNA (concentration of 1.0 × 10^4^ CFU/mL) was extracted and used as the template for the LAMP reaction, and the products were observed by fluorescence dye, 3% agarose gel electrophoresis, and LFD analysis. Results were considered positive when the fluorescent dyes were green, while the negative control was orange (Fig. 2A). The agarose gel electrophoresis showed obvious trapezoidal bands (Fig. 2B), while the negative control had no trapezoidal band. LAMP-LFD results of positive *M. ovipneumoniae* template appeared in two red strips, respectively located in the test line (T) and control line (C), and negative control was only in one control line (C) a red strip (Fig. 2C). The results showed that the LAMP-LFD assay was effective and feasible.

### Specificity and repeatability of LAMP-LFD

**Fig. 3:**
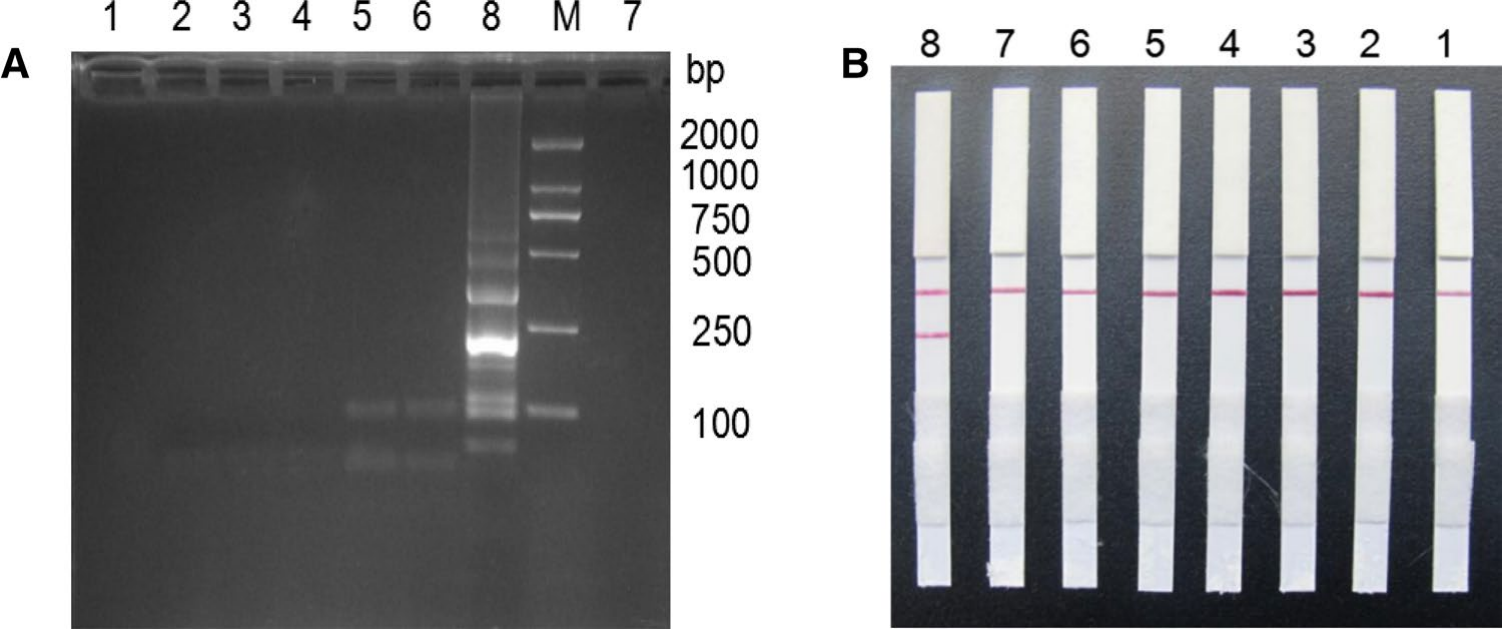
Specific analysis results of LAMP-LFD. **A** LAMP agarose gel electrophoresis. **B** LAMP-LFD detection. M: DNA Marker; 1: using sterile water as the template for negative control; 2–7: the genomic DNA of *Escherichia coli, Staphylococcus aureus, Salmonella pullorum, M. Bovis, M. hyopneumoniae* and *M. mycoides* subsp. *Capri* were used as template. 8: the genomic DNA of *M. ovipneumoniae* was used as the template

**Fig. 4:**
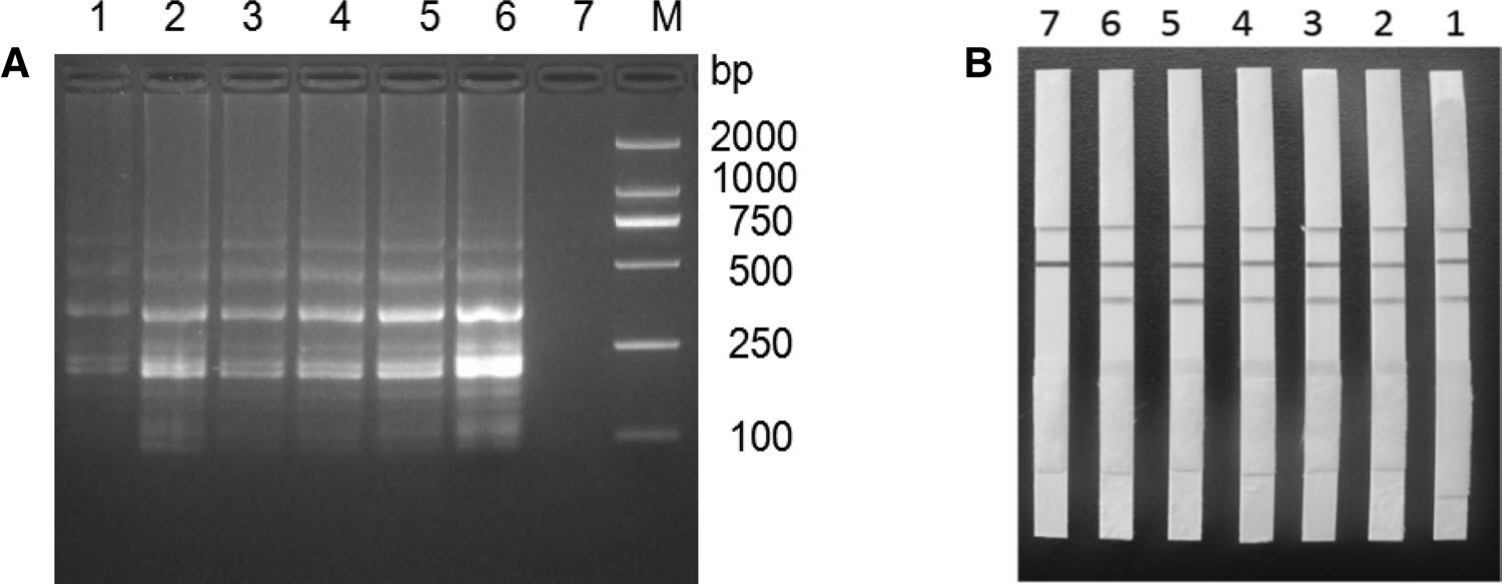
Repeatability analysis of LAMP-LFD. **A** LAMP agarose gel electrophoresis; **B** LAMP-LFD assay. M: DNA Marker; 1–6: the genomic DNA of *M. ovipneumoniae* (1.0 × 10^4^ CFU/mL) was used as the template; 7: negative control

Based on the best LAMP reaction system and conditions, biotin-labeled primers in *EF-TU*FIP LAMP reaction, with four strains test strains and *M. ovipneumoniae* were used as templates to carry out specificity validation. The results showed that with *M. ovipneumoniae* genomic DNA as a template, LAMP product after electrophoresis had the same characteristic trapezoidal strips as the LAMP products and probe *EF-TU*-HP hybrid. Moreover, hybrid products on the LFD strip line position had a clear red strip, indicating that the test result was positive. Meanwhile the other detectable microbial LAMP products were negative, and the *EF-TU*-HP hybrid product in LFD position was not present in any strip on the line, indicating that the established *M. ovipneumoniae* LAMP-LFD detection method had good specificity (Table 2; Fig. 3). When the *M. ovipneumoniae* genomic DNA was repeated as a template, the results were also positive, showing that the test method had good repeatability (Fig. 4).

**Table 2:** Specific analysis results of LAMP-LFD

Bacteria	Strains	Result
*Escherichia coli*	EC-xj 01	−
*Staphylococcus aureus*	CVCC 1885	−
*Salmonella pullorum*	CVCC 1791	−
*M. bovis*	CGMCC 13295	−
*M. hyopneumoniae*	CGMCC 8011	−
*M. mycoides* subsp. *Capri*	CVCC 3011	−
*M. ovipneumoniae*	CVCC 384	+

“+” means positive; “−” means negative

### Sensitivity of LAMP-LFD

**Fig. 5:**
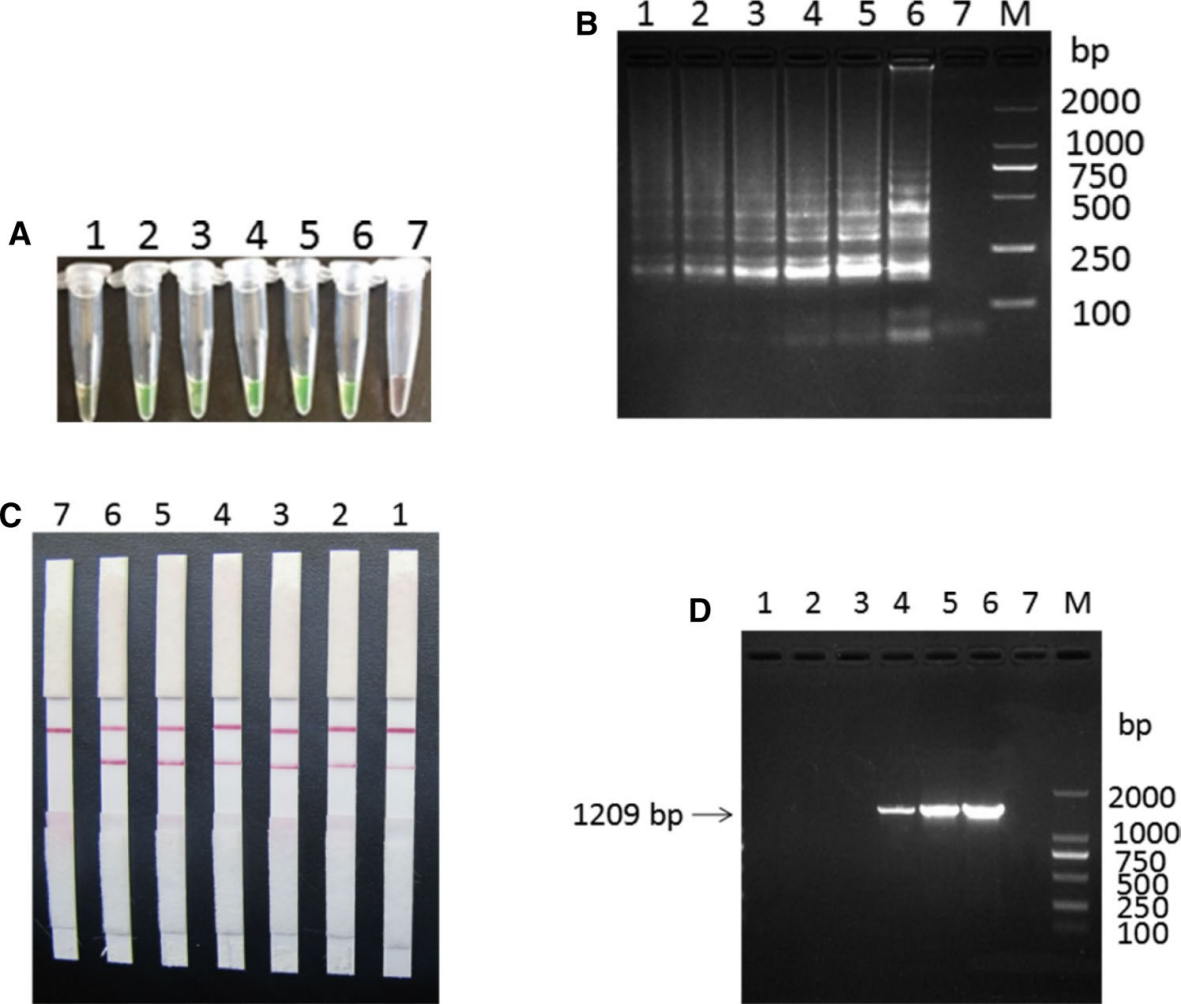
Sensitivity analysis of the *M. ovipneumoniae* LAMP-LFD. **A** calcium-visual LAMP sensitivity test. **B** LAMP agrogel electrophoresis method for sensitivity test. **C** Sensitivity test of LAMP-LFD method; **D** Sensitivity test of PCR assay. M: DNA Marker; 1–6: different concentrations of positive standard: 1 × 10^2^ CFU/mL; 1 × 10^3^ CFU/mL; 1 × 10^4^ CFU/mL; 1 × 10^5^ CFU/mL; 1 × 10^6^ CFU/mL; 1 × 10^7^ CFU/mL; 7: negative control

Next, we performed sensitivity analysis of LAMP-LFD using different concentrations of *M. ovipneumoniae* genomic DNA as a template. LAMP-LFD reaction results showed that with the calcein-visual LAMP (Fig. 5A), LAMP agarose gel electrophoresis (Fig. 5B), and LAMP-LFD (Fig. 5C) to detect the sensitivity, the minimum detectable concentration was 1.0 × 10^2^ CFU/mL. The minimum detectable method of PCR was 1.0 × 10^5^ CFU/mL (Fig. 5D), which showed that the sensitivity of LAMP-LFD was 1000 times that of the conventional PCR assay.

### Clinical application detection of *M. ovipneumoniae*

We next determined the clinical application using the established conditions of the LAMP-LFD assay as compared to pathogen separation (gold standard) in 50 suspected *M. ovipneumoniae*-infected sheep lesions by lung tissue genomic DNA testing. As shown on Table 3, LAMP-LFD assay identified 43 as positive for *M. ovipneumoniae*, was 100% consistent with the results of the gold standard method. By comparison, conventional PCR assay detected 41 positive samples, which indicated the detection rate was 82%, was 95.35% consistent with the results of the gold standard method, showing that LAMP-LFD could be preliminarily used in the detection of clinical pathogens.

**Table 3:** Detection results of clinical samples using gold standard, LAMP-LFD and the PCR assay

Detection method	Positive number (case)	Negative number (case)	Positive rate (%)	Coincidence (%)
Pathogen separation (gold standard)	43	7	86	/
LAMP-LFD	43	7	86	100
PCR	41	9	82	95.35

## Discussion

In recent years, with the rise in socioeconomic status and quality of life, there has been a higher demand for lamb and wool products, which has increased sheep farming and husbandry (Di et al. 2015). However, the incidence of *M. ovipneumoniae* targeting sheep has concordantly been on the rise, with epidemic outbreaks reported recently (Bottinelli et al. 2017; Wolff et al. 2018). Therefore, there is a need to establish a rapid, simple, and reliable detection method for *M. ovipneumoniae*. In this study, a fast and efficient LAMP-LFD assay for detecting *M. ovipneumoniae* was established by combining LAMP and LFD technology. The method is simple, with a low requirement in experimental conditions, and results are presented visually. The advantages of this method are irreplaceable by other molecular biological methods. LAMP-LFD detection needn’t calcein, compared with the LAMP method; thus it not consider the calcein transport issues, greatly reduces the economic losses caused by transportation process. And water will be polluted by calcein, sewage treatment is more troublesome, causing certain economic losses. The sensitivity of *M. ovipneumoniae* samples was 5 × 10^2^ CFU/mL, which was 1000 times higher than conventional PCR methods. In this study, *M. ovipneumoniae* standard strain Y98 was selected for analysis, and the LAMP gene amplification of the *EF-TU* gene showed positive results, while other pathogenic strains showed negative results. Therefore, this method has good specificity. Various components of the LAMP-LFD reaction were optimized: inside and outside primer concentration ratios and LAMP reaction components, i.e., Mg^2+^ concentration, dNTPs concentration, temperature, and reaction time. Once the reaction system and the optimal reaction conditions were determined, the results showed that LAMP could effectively complete the amplification within 60 min (Bai et al. 2011), which is faster than other methods. Moreover, the clinical application of the LAMP-LFD assay was tested using 50 suspected *M. ovipneumoniae*-infected sheep lesions, LAMP-LFD assay identified 43 as positive for *M. ovipneumoniae*; the parallel PCR identified 41 as positive. LAMP-LFD was 100% consistent with the results of the gold standard method, while PCR was 95.35% consistent, ang it takes about 2.5 h to complete all the steps, pathogen separation (gold standard) takes more time, implying that the LAMP-LFD assay can be applied to the clinical detection of pathogens.

Current detection methods of *M. ovipneumoniae* include PCR, indirect hemagglutination test, and ELISA (Song et al. 2014; Maksimović et al. 2017; Beser et al. 2012). These methods all have high sensitivity and accuracy but require expensive instruments and have tedious operation steps; thus, it may not be feasible for economically disadvantaged farms and clinics. LAMP-LFD technology has many advantages, such as its simplicity and speed, lack of expensive instruments and equipment, and accuracy of its results and real-time visualization. LAMP-LFD has been applied to food detection, vaccine diagnosis and disease diagnosis by many researchers (Yang et al. 2016; Deng et al. 2015; Rigano et al. 2014; Khunthong et al. 2013; Sun et al. 2014).

In this study, we used the *M. ovipneumoniae EF-TU* gene as the detection target and established that the LAMP-LFD assay has strong specificity, high sensitivity, and visual results. Our experimental conditions were optimized at 60 °C under the condition of constant temperature for 60 min, which could complete the detection of *M. ovipneumoniae*. Furthermore, the requirements for instruments and specific experimental conditions were lower, the sensitivity was 1.0 × 10^2^ CFU/mL, and the method could detect *M. ovipneumoniae* on-site. Results were revealed by the color strip on the dipstick, which means that the procedure does not require special equipment for detection, increasing the amplification efficiency, convenience, and practicality and avoiding the nonspecific amplification products that often lead to false positives. Therefore, the establishment of this method to detect *M. ovipneumoniae* provides a practical technology and lays the foundation for the development of a clinical kit, which can be used as a new *M. ovipneumoniae* detection method in basic-level livestock units.

## Electronic supplementary material

Below is the link to the electronic supplementary material.

Supplementary material 1 (DOCX 5827 KB)
