# Supplementary material for: Loop-mediated isothermal amplification-lateral-flow dipstick (LAMP-LFD) to detect Mycoplasma ovipneumoniae
Source: World J Microbiol Biotechnol. 2019 Jan 30;35(2):31. doi: 10.1007/s11274-019-2601-5 (PMC6353813; doi:10.1007/s11274-019-2601-5)
Supplement: Supplementary file 1 — Supplementary material 1 (DOCX 5827 KB) [file 11274_2019_2601_MOESM1_ESM.docx]

**
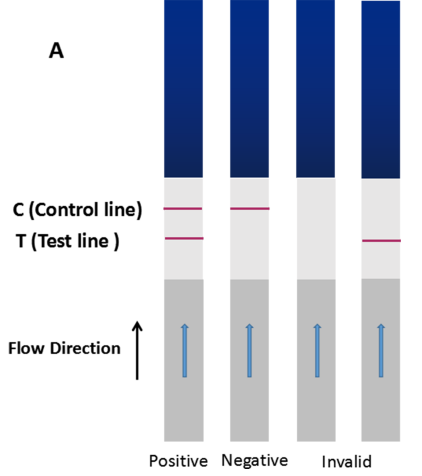

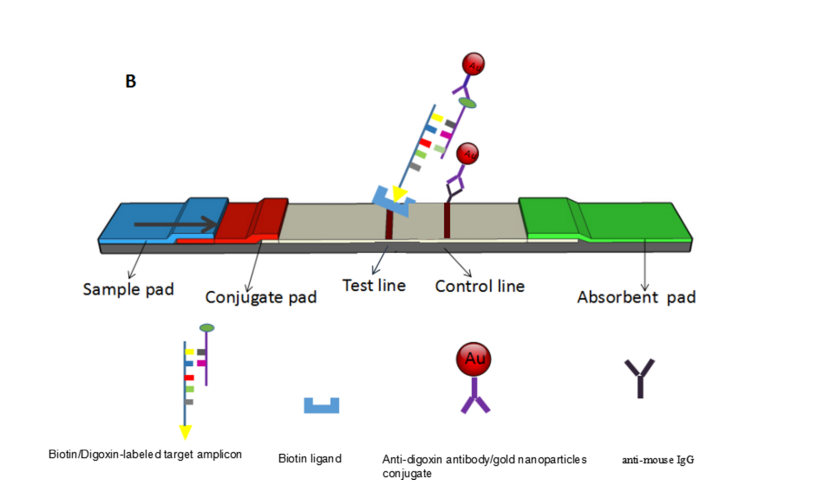
**

**Extended Data Fig. S1 LAMP-LFD mechanism schematic representation.** (A) Results evaluation using LFD. The determination criterion is positive result when the control line (C) and test line (T) both have the red strip. The result is negative when only the control line has the red strip. The dipstick is invalid when only the test line (T) has the red strip or both the control line (C) and test line (T) lack the red strip. (B) The schematic diagram of how the hybrid product of LAMP amplification captures the antibody on LFD. A biotin LAMP product hybridized with a digoxin-labeled DNA probe and complexed with a gold-labeled anti-digoxin antibody. This hybridization product is trapped by a biotin ligand and bound to a lateral flow dipstick, forming an immune complex, bound to the test line (T). Non-hybridized digoxin-labeled probes passed through the test line (T) and bound to the sheep anti-mouse IgG antibody control line (C).


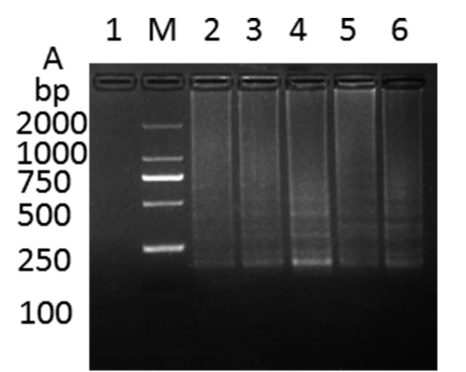

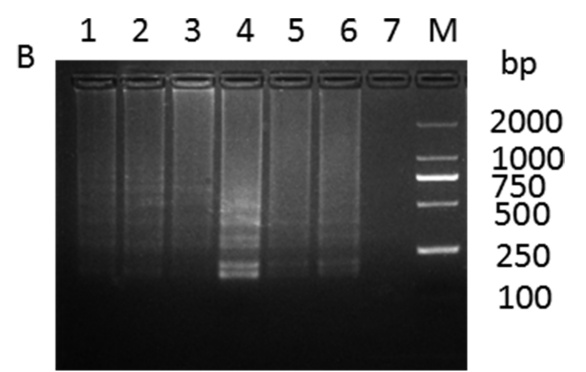


**Extended Data Fig. S2 Optimization of reaction temperature and time.** (A) optimization of reaction temperature, M: DNA Marker; 1: negative control; 2-6: reaction temperature of 58°C, 59°C, 60°C, 61°C and 61°C. (B) optimization of reaction time, M: DNA Marker; 1-6 is 30 min, 40 min, 50 min, 60 min, 70 min and 80 min respectively; 7: negative control.


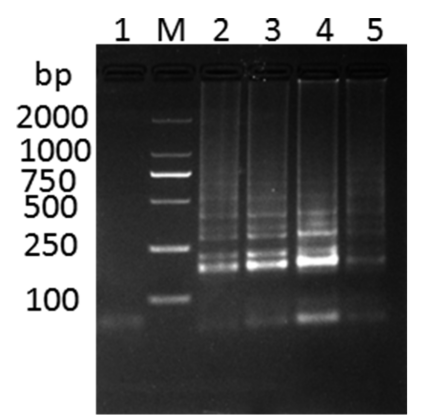


**Extended Data Fig. S3 Optimization of primer concentration ratio in the reaction system.** M: DNA Marker; 1: negative control; 2-5: the primer ratio is 1:1, 2:1, 3:1, 4:1.


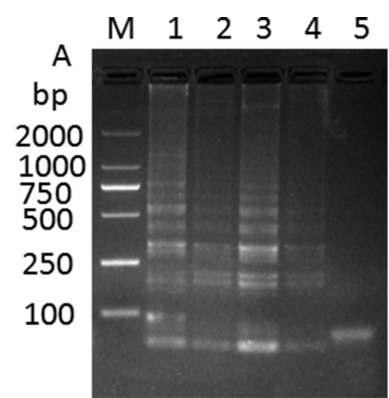

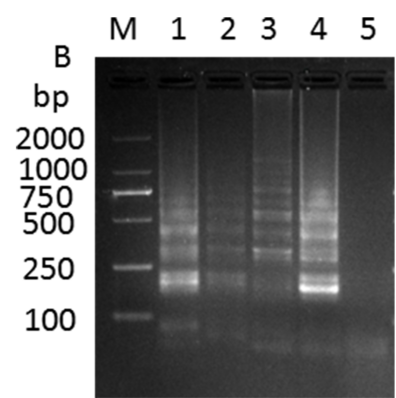


**Extended Data Fig. S4 Optimal reaction system.** (A) optimization of MgSO_4_ in the reaction system, M: DNA Marker; 1-4: The volume of MgSO_4_ was 1.0 μL, 1.5 μL, 2.0 μL, 2.5μL. 5: negative control. (B) optimization of dNTPs in the reaction system, M: DNA Marker; 1-4: The volume of dNTPs was 1.0 μL, 1.5 μL, 2.0 μL, 2.5 μL. 5: negative control.
